# Gankyrin activates the hedgehog signalling to drive metastasis in osteosarcoma

**DOI:** 10.1111/jcmm.16576

**Published:** 2021-06-05

**Authors:** Chongchong Wang, Juehua Jing, Xuyang Hu, Shuisheng Yu, Fei Yao, Ziyu Li, Li Cheng

**Affiliations:** ^1^ Department of Oncology The Fourth Affiliated Hospital of Anhui Medical University Hefei China; ^2^ Department of Orthopaedics The Second Affiliated Hospital of Anhui Medical University Hefei China; ^3^ School of pharmacy Anhui Medical University Hefei China

**Keywords:** gankyrin, hedgehog signalling, metastasis, osteosarcoma

## Abstract

Gankyrin is a regulatory subunit of the 26‐kD proteasome complex and promotes the occurrence and progression of many malignancies. However, the role of gankyrin in osteosarcoma (OS) metastasis remains unclear. Hedgehog signalling has been shown to regulate stem cell homeostasis and cancer metastasis, but the mechanisms that activate this pathway in OS are still poorly understood. Here, a series of in vitro and in vivo assays were carried out to explore the function and mechanism of gankyrin regulating Hedgehog signalling in OS. We demonstrated that gankyrin promotes migration, invasion and regulates the expression of some stemness factors by up‐regulating Gli1 in OS. Importantly, our data showed an interaction between gankyrin and Gli1. Moreover, gankyrin suppresses the ubiquitin‐mediated degradation of Gli1 protein in OS. Gankyrin also significantly promotes the lung metastasis of OS in vivo. Our findings suggest that gankyrin drives metastasis and regulates the expression of some stemness factors in osteosarcoma by activating Hedgehog signalling, indicating that drug screening for compounds targeting gankyrin may contribute to the development of novel and effective therapies for OS.

## 1 INTRODUCTION

Osteosarcoma (OS) is a primary malignant bone tumour that commonly affects children and adolescents.^1^, ^2^ Despite progress in chemotherapy and surgical approaches, the survival rate of OS is still unsatisfactory because of frequent lung metastasis.^3^ Hence, a deeper understanding of the underlying molecular mechanisms of OS progression and the identification of molecular targets that are dependent on complex gene regulation networks are urgently needed.

Gankyrin, also named PSMD10, is one of the regulatory subunits of the 26‐kD human proteasome complex.^4^ Our previous study showed that gankyrin is an oncoprotein that is frequently up‐regulated in OS.^5^ In addition, increasing evidence suggests that gankyrin is involved in the occurrence and progression of various tumours.^6^, ^7^, ^8^ Nevertheless, the potential role of gankyrin in OS metastasis is still unclear. Cancer stem cells (CSCs) have been demonstrated to support cancers by managing non‐genetic and genetic factors that regulate metastasis and cancer microenvironment maintenance.^9^, ^10^ However, whether gankyrin regulates cancer stemness remains unknown.

Hyperactive Hedgehog signalling has also been associated with many malignancies, such as OS and lung, colon, breast and pancreatic cancer.^11^, ^12^, ^13^, ^14^, ^15^, ^16^ Functionally, Hedgehog signalling regulates cancer stemness and metastasis.^17^, ^18^ Gli1 is the major transcriptional activator in Hedgehog signalling and has been shown to mediate the activation of several Hedgehog target genes, including *Hhip*, *Ptch1*, *Cyclin D1* and *Snail*.^18^, ^19^, ^20^ In the present study, we found that gankyrin promotes migration, invasion and regulates the expression of some stemness factors by up‐regulating Gli1 in OS. An association between gankyrin and Gli1 was confirmed. Furthermore, gankyrin suppresses the ubiquitin‐mediated degradation of Gli1 protein in OS. Importantly, gankyrin promotes the lung metastasis of OS in vivo. Our data indicate the possibility of targeting gankyrin‐Gli1 regulation for the treatment of OS.

## 2 METHODS

### 2.1 Plasmids and reagents

Human gankyrin cDNA fragments, short hairpin RNA (shRNA) targeting human gankyrin and shRNA targeting Gli1 were obtained from GenePharma. Lentiviral vectors expressing gankyrin, shRNA against gankyrin (shGankyrin), shRNA against Gli1 (shGli1) and the corresponding controls were purchased from Servicebio. Anti‐gankyrin (3A6C2) was purchased from Santa Cruz Biotechnology. Anti‐β‐tubulin (Ag0117), anti‐CD133 (Ag13327), anti‐OCT4 (Ag1794), anti‐ubiquitin (Ag0260) and anti‐GAPDH (Ag0766) antibodies were obtained from Proteintech. Anti‐Nanog (GB11331) was purchased from Servicebio (Wuhan). Anti‐Flag (T0003), anti‐Gli1 (DF7523) and anti‐PTCH1 (AF5202) were from Affinity. Anti‐rabbit IgG‐peroxidase (A0545) and anti‐mouse IgG‐peroxidase (A4416) were purchased from Sigma. Alexa Fluor 594 anti‐rabbit IgG (A‐21207) and Alexa Fluor 488 anti‐mouse IgG (A‐21202) were purchased from Invitrogen.

### 2.2 Human OS samples

Human OS tissues were obtained from 56 OS patients who underwent tumour resection combined with neoadjuvant chemotherapy at the First Affiliated Hospital of Anhui Medical University, Second Affiliated Hospital of Anhui Medical University and Anhui Provincial Hospital. Patients were recruited with a scientific ethics consent form, and all processes were approved by the Ethics Committee of the Second Affiliated Hospital of Anhui Medical University (No. 20170240).

### 2.3 Cell culture, transfection, and viral infection

U2OS and MG63 cells were obtained from the Shanghai Cell Bank of the Chinese Academy of Sciences. Cells were grown in RPMI 1640 medium (Gibco) supplemented with 10% foetal bovine serum (Gibco) at 37°C with 5% CO_2_. Transfection was carried out utilizing Lipofectamine 2000 reagent (Invitrogen) following the manufacturer's instructions. Viral infection was utilized to produce stable cells. Briefly, MG63 and U2OS cells were infected with lentiviral vectors expressing gankyrin + shGli1, gankyrin, or the corresponding control supplemented with 8 μg/mL polybrene (Sigma). The virus‐containing medium was removed and replaced with normal medium after 24 hours. Transfection efficiency was detected through Western blot analysis at 48 hours after treatment.

### 2.4 Co‐immunoprecipitation (Co‐IP) and Western blot analyses

Cells were lysed in a buffer containing 25 mmol/L Tris‐HCl, 1 mmol/L dithioerythritol, 150 mmol/L NaCl, 0.5 mmol/L phenylmethylsulphonyl fluoride, 2 mmol/L ethylenediaminetetraacetic acid, 0.1% Nonidet P‐40 and protease inhibitor and phosphatase inhibitor cocktails (Roche). Cell lysates were incubated with Protein A/G magnetic beads (Thermo Fisher Scientific) and anti‐gankyrin antibody overnight at 4°C. The protein complexes were washed and subjected to Western blot analysis. Then, cell lysates or Co‐IP samples were separated by SDS‐PAGE, transferred to PVDF membranes, blocked using PBS/Tween‐20 (PBST) containing 5% nonfat milk, incubated with the indicated primary and secondary antibodies, and visualized with an enhanced chemiluminescence (ECL) system.

### 2.5 Wound healing and Transwell invasion assays

Different groups of OS cells were seeded into a 6‐well plate and cultured overnight. When cells reached a density of more than 90%, wounds were scratched with a 20‐μL pipette tip, and then suspended cells were removed. Micrographs were taken at 0 hour and 24 hours. For the invasion assay, Transwell chambers were coated with Matrigel overnight. Then, 2 × 10^4^ cells without serum were placed into the upper chamber, and RPMI 1640 medium containing 10% foetal bovine serum was added to the lower chamber. Cells in the upper chamber were removed using cotton swabs at 24 hours. Then, cells in the lower chamber were fixed using 4% paraformaldehyde, stained with 0.1% crystal violet and photographed.

### 2.6 Real‐time quantitative (qRT) PCR analysis

Total RNA was extracted utilizing TRIzol reagent (Invitrogen), and cDNA was obtained using the PrimeScript RT reagent kit (TaKaRa). Real‐time PCR was carried out using the SYBR Green PCR Master Mix kit (TaKaRa) in an ABI Step‐One system. β‐Actin was used for sample normalization. The primer sequences are listed in Table S1.

### 2.7 Immunofluorescence (IF) assay

Cells were cultured on cover slips in 6‐well plates until they reached 60% confluence and were then fixed with 4% paraformaldehyde for 10 minutes. Afterwards, the cells were permeabilized with 0.1% Triton X‐100 for 2 minutes. Then, the cells were blocked with 5% bovine serum albumin for 30 minutes at 37°C and incubated with anti‐gankyrin and anti‐Gli1 antibodies overnight at 4°C. Then, the cells were incubated with Alexa Fluor 594 anti‐rabbit IgG and Alexa Fluor 488 anti‐mouse IgG antibodies for 1 hour at 37°C. The cells were then stained with 4’6‐diamidino‐2‐phenylindole (DAPI) and photographed.

### 2.8 Immunohistochemistry (IHC) analysis

Paraffin sections were deparaffinized, treated with citrate buffer and incubated with hydrogen peroxide. Then, sections were blocked with 10% goat serum for 30 minutes at 37°C and incubated with anti‐gankyrin or anti‐Gli1 antibodies overnight at 4°C. Afterwards, sections were incubated with a biotin‐labelled secondary antibody for 1 hour at 37°C and then with streptavidin‐conjugated horseradish peroxidase. Sections were then developed utilizing diaminobenzidine and counterstained using haematoxylin. A staining intensity scoring system (values 0‐6) was designed by adding the staining intensity scores (0, negative; 1, weak; 2, moderate; 3, strong) and the staining extent scores (0, 0% of stained cells; 1, <5% of stained cells; 2, 5%‐50% of stained cells; 3, >50% of stained cells). The immunostaining was categorized into two groups: low, scores 0 and 2; high, scores 3‐6.

### 2.9 In vivo metastasis model

All animal experiments were carried out according to the Guide for the Care and Use of Laboratory Animals and were approved by the Institutional Animal Care and Use Committee of Anhui Medical University (No. LLSC20170035). U2OS cells stably transfected with lentiviral vectors expressing gankyrin + shGli1, gankyrin or the corresponding control (3 × 10^5^ cells in 25 μL PBS, five mice per group) were intravenously injected into the tail veins of BALB/c nude mice at 4 weeks of age. After 9 weeks, the mice were sacrificed, the lungs were weighed, sectioned and stained with haematoxylin and eosin (H&E), and the number of lung metastases was counted.

### 2.10 Statistical analysis

SPSS 19.0 software was used for the statistical analysis. The data are presented as the means ± SD, and at least three independent experiments were performed. Comparisons between groups were analysed with Student's *t* test. Overall survival was determined utilizing the log rank test and is shown as Kaplan‐Meier curves. Spearman's correlation analysis was used to explore the correlation between gankyrin and Gli1 levels. Differences were considered significant when the *P* value was less than 0.05. ns, statistically non‐significant, *P* ≥ .05; *P < .05; **P < .01; and ***P < .001.

## 3 RESULTS

### 3.1 Gankyrin promotes migration, invasion and regulates the expression of some stemness factors in OS

**FIGURE 1:**
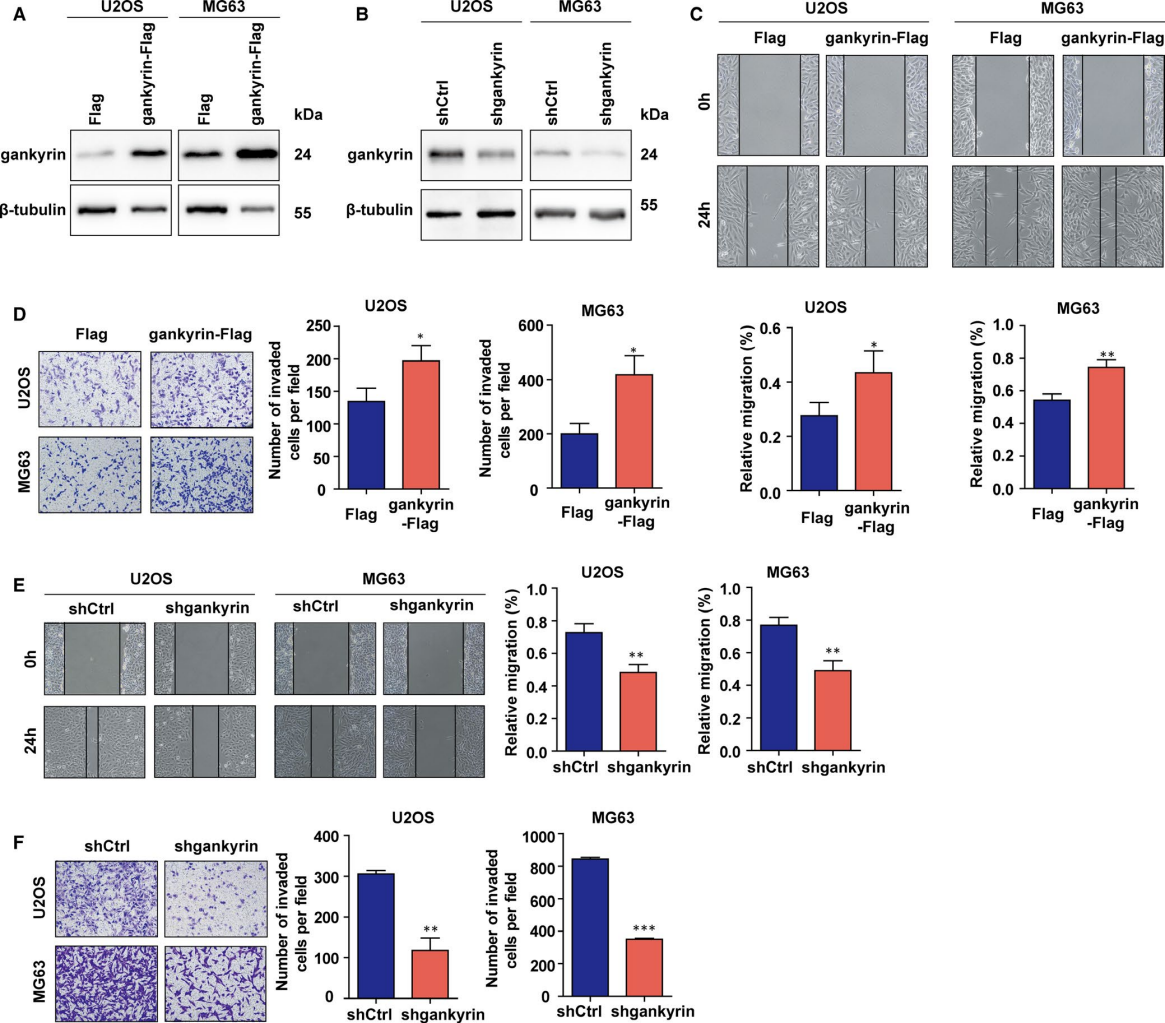
Gankyrin promotes migration and invasion in OS in vitro. A, Induction of gankyrin in U2OS and MG63 cells was confirmed by Western blot analysis. B, Knockdown of gankyrin in U2OS and MG63 cells was confirmed via Western blot analysis. C, Induction of gankyrin significantly increased OS cell migration ability, as shown by wound healing assays. *P < .05; **P < .01. D, Up‐regulated gankyrin remarkably promoted OS cell invasion, as shown by Transwell invasion assays. *P < .05. E, Knockdown of gankyrin reduced OS cell migration ability. **P < .01. F, Knockdown of gankyrin inhibited OS cell invasion. **P < .01; ***P < .001

**FIGURE 2:**
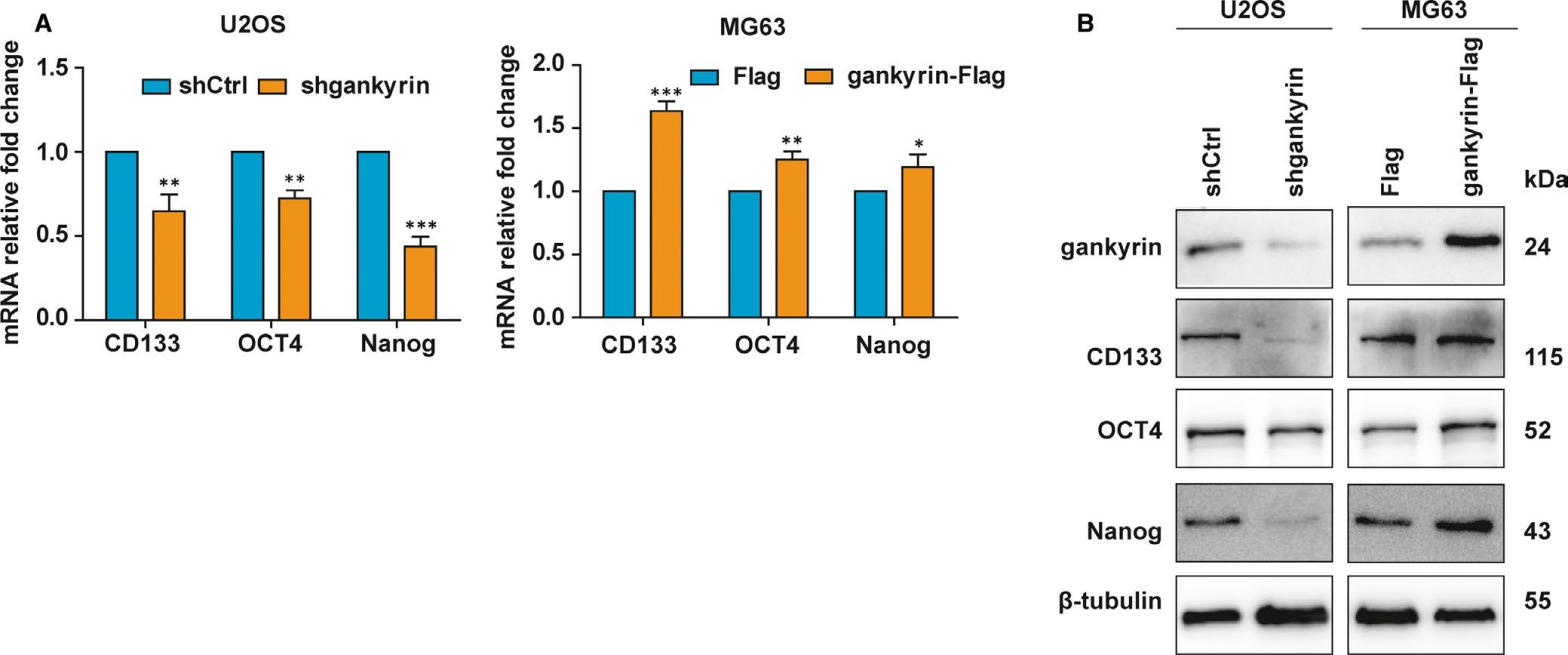
Gankyrin regulates the expression of some stemness factors in OS. A, Gankyrin knockdown in U2OS cells reduced the mRNA levels of stem cell markers, including CD133, OCT4 and Nanog, as shown by qRT‐PCR analysis, whereas overexpression of gankyrin in MG63 cells induced these markers at the mRNA level. *P < .05; **P < .01; and ***P < .001. B, Gankyrin knockdown in U2OS cells reduced the protein levels of stem cell markers, including CD133, OCT4 and Nanog, as shown by Western blot analysis, whereas overexpression of gankyrin in MG63 cells induced the expression of markers at the protein level

To investigate the roles of gankyrin in OS cell migration and invasion, we performed wound healing assays and Transwell invasion assays. Overexpression of gankyrin in U2OS and MG63 cells was carried out using a flag‐tagged gankyrin plasmid, while knockdown of gankyrin was established with a short hairpin RNA (Figure 1A, B; Figure S1). The data showed that overexpression of gankyrin significantly promoted OS cell migration and invasion (Figure 1C, D), whereas gankyrin knockdown inhibited cell migration and invasion (Figure 1E, F). Previous studies revealed that CSCs play vital roles in OS metastasis.^21^, ^22^ Moreover, gankyrin may influence stem cell behaviour by controlling the expression of diverse stemness factors.^23^, ^24^ We thus detected the influence of gankyrin on OS stemness by examining the expression of CSC markers. Obviously, gankyrin knockdown in U2OS cells resulted in a marked reduction in stem cell markers, including CD133, OCT4 and Nanog, at both the mRNA and protein levels, whereas gankyrin overexpression in MG63 cells led to up‐regulation of these stem factors (Figure 2A, B). These results indicate that gankyrin promotes migration, invasion and regulates the expression of some stemness factors in OS.

### 3.2 Gankyrin promotes invasion, migration and regulates the expression of some stemness factors by up‐regulating Gli1

**FIGURE 3:**
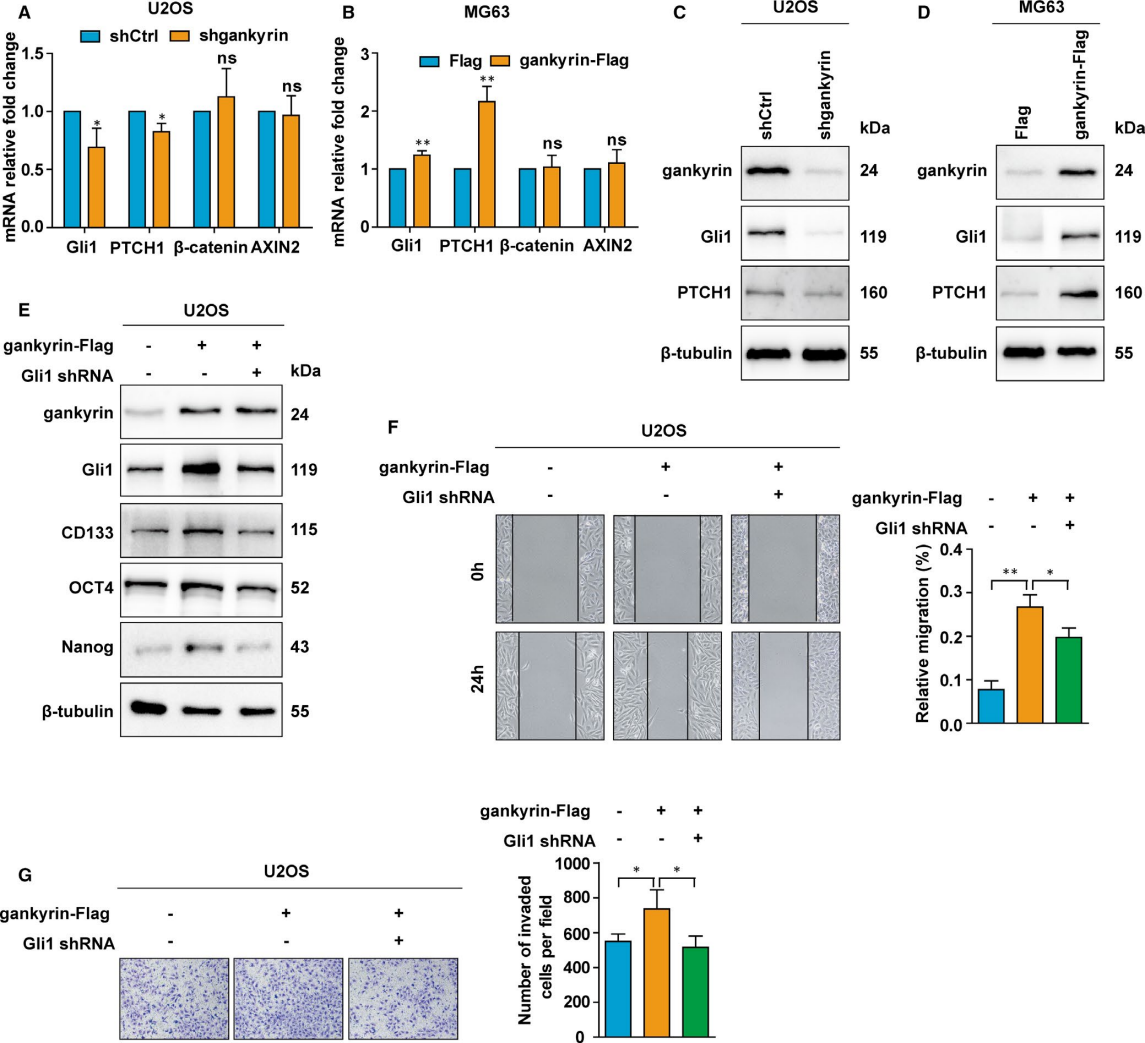
Gankyrin promotes invasion, migration and regulates the expression of some stemness factors by up‐regulating Gli1. A, Gankyrin knockdown in U2OS cells reduced the mRNA expression of Gli1 and Ptch1 but not of β‐catenin and AXIN2, as shown by qRT‐PCR analysis. ns, *P* ≥ .05; *P < .05. B, Overexpression of gankyrin in MG63 cells increased the mRNA expression of Gli1 and Ptch1 but not of β‐catenin and AXIN2. ns, *P* ≥ .05; **P < .01. C, Gankyrin depletion in U2OS cells diminished the protein levels of Gli1 and PTCH1, as detected by western blot analysis. D, Ectopic expression of gankyrin in MG63 cells induced the protein levels of Gli1 and PTCH1. E, The up‐regulation of stem cell markers, including CD133, OCT4, and Nanog, following gankyrin overexpression was attenuated by Gli1 shRNA in U2OS cells, as detected by Western blot analysis. F, The increase in cell migration with gankyrin overexpression was attenuated by Gli1 shRNA in U2OS cells, as shown by wound healing assays. *P < .05; **P < .01. G, The increase in cell migration with gankyrin overexpression was attenuated by Gli1 shRNA in U2OS cells, as shown by Transwell invasion assays. *P < .05

It has been demonstrated that the Hedgehog and Wnt signalling pathways are involved in the regulation of tumour invasion, migration and stemness.^17^, ^25^, ^26^, ^27^, ^28^ We thus assessed the potential effect of gankyrin on these pathways. Knockdown of gankyrin reduced the mRNA expression of several key Hedgehog signalling genes, such as Gli1 and PTCH1, but not of β‐catenin and AXIN2, which are involved in the Wnt signalling pathway (Figure 3A). In contrast, gankyrin overexpression increased the mRNA expression of Gli1 and PTCH1 (Figure 3B). Consistently, gankyrin depletion distinctly diminished the protein levels of Gli1 and PTCH1, while ectopic expression of gankyrin induced these levels (Figure 3C, D). Given that Gli1 is the final and most crucial effector of Hedgehog signalling,^19^ we next evaluated whether the induction of metastasis and stemness by gankyrin is mediated by Gli1. The results showed that the up‐regulation of stem cell markers, including CD133, OCT4 and Nanog, following gankyrin overexpression was suppressed by Gli1 shRNA (Figure 3E). In addition, the increased cell migration and invasion by gankyrin overexpression were rescued by Gli1 shRNA (Figure 3F, G). Moreover, considering that Gli1 is activated upon its nuclear translocation, we next assessed the effect of gankyrin on nuclear localization of Gli1. Nuclear localization of Gli1 was not affected by gankyrin in U2OS or MG63 cells (Figure S2). These findings indicate that gankyrin promotes invasion, migration and regulates the expression of some stemness factors by up‐regulating Gli1.

### 3.3 Gankyrin interacts with Gli1

**FIGURE 4:**
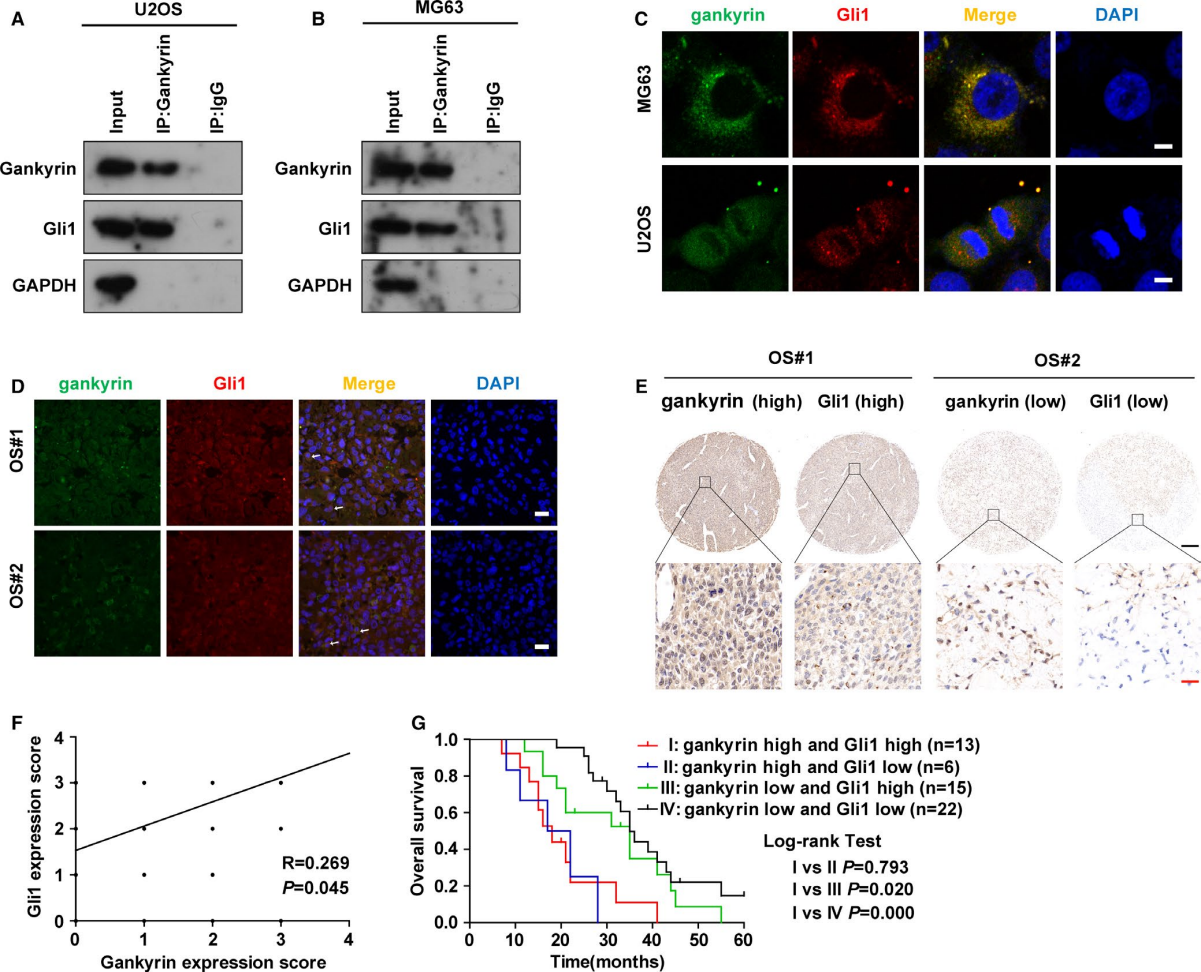
Association between gankyrin and Gli1. A, Gankyrin interacted with Gli1 in U2OS cells, as detected by a Co‐IP assay. Gankyrin was immunoprecipitated with anti‐gankyrin antibodies, and Co‐IP of Gli1 was performed with anti‐Gli1 antibodies. IgG was used as the negative control. B, Gankyrin interacted with Gli1 in MG63 cells. Gankyrin was immunoprecipitated with anti‐gankyrin antibodies, and Co‐IP of Gli1 was performed with anti‐Gli1 antibodies. IgG was used as the negative control. C, Co‐localization of gankyrin and Gli1 in U2OS and MG63 cells was revealed via immunofluorescence assays. Scale bar, 10 μm. D, Co‐localization of gankyrin and Gli1 in human OS samples. Representative images in two human OS tissues are shown. The white arrows indicate co‐localization of gankyrin and Gli1. Scale bar, 30 μm. E, Representative images of gankyrin and Gli1 IHC staining in human OS samples revealed that gankyrin is associated with Gli1. F, The association between gankyrin and Gli1 expression in 56 human OS samples was assessed using Spearman's correlation analysis. G, The 5‐year overall survival rates of OS patients with different gankyrin and Gli1 levels are shown in Kaplan‐Meier curves, and the significance of differences between groups was determined utilizing the log rank test

To better determine the molecular mechanism underlying gankyrin‐mediated regulation of the Hedgehog signalling pathway, we carried out Co‐IP assays and found that gankyrin interacts with Gli1 in U2OS (Figure 4A) and MG63 (Figure 4B) cells. Moreover, immunofluorescence assays showed that gankyrin and Gli1 co‐localized in both OS cells (Figure 4C) and OS tissues (Figure 4D). To determine the clinical relevance of gankyrin interacting with Gli1, we next performed IHC staining of 56 OS specimens and found a positive association between gankyrin and Gli1 expression in OS samples (Figure 4E, F). Notably, the patients with co‐elevated levels of gankyrin and Gli1 exhibited worse overall survival than patients who had only up‐regulated Gli1 levels (*P* = .020) and those who did not have elevated gankyrin or Gli1 levels (*P* = .000) (Figure 4G). These data indicate a close relationship between gankyrin and Gli1 in OS.

### 3.4 Gankyrin suppresses the ubiquitin‐mediated degradation of Gli1 protein

**FIGURE 5:**
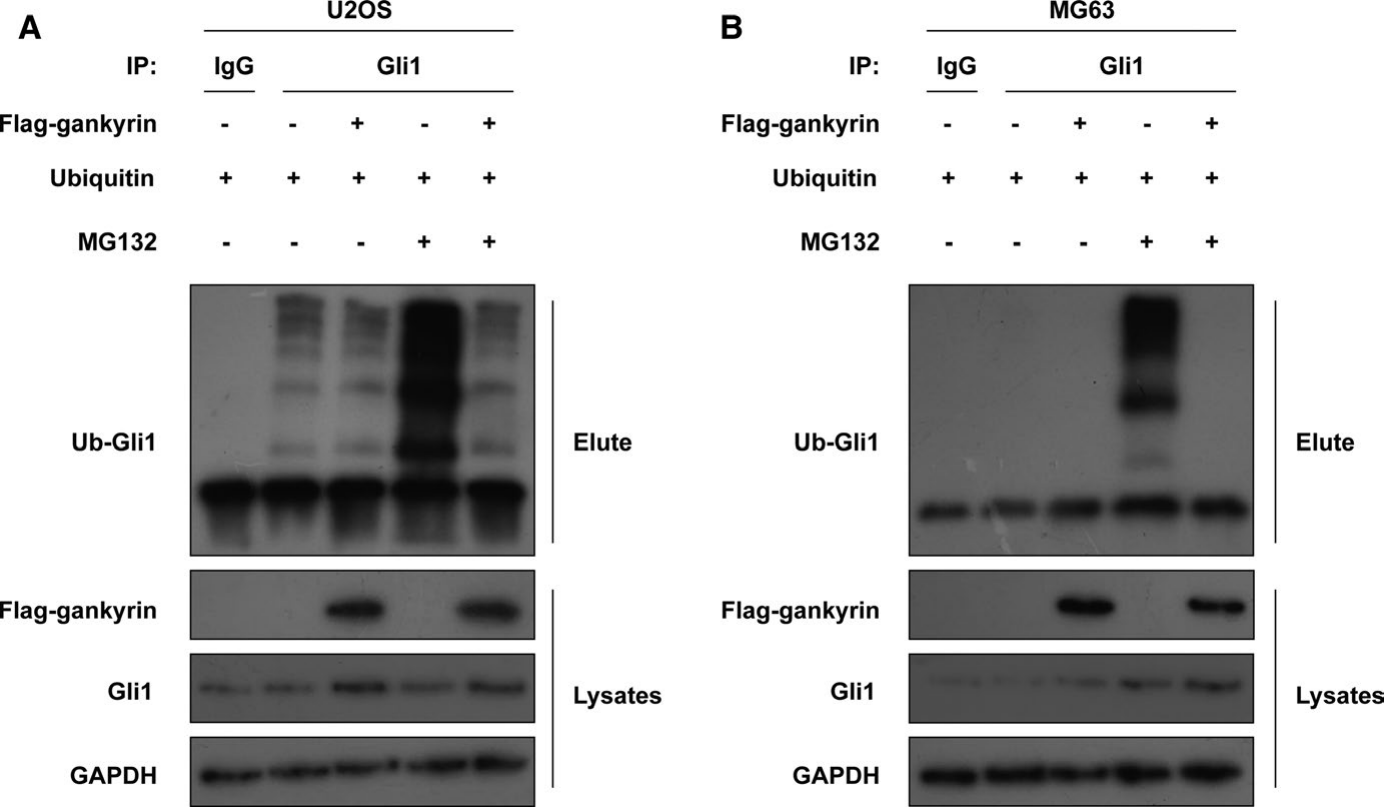
Gankyrin suppresses the ubiquitin‐mediated degradation of Gli1 protein. A, U2OS cells were treated with MG132, and cellular lysates were immunoprecipitated with anti‐Gli1 antibody. Gankyrin reduced Gli1 ubiquitination and induced Gli1 protein expression. B, MG63 cells were treated with MG132, and cellular lysates were immunoprecipitated with anti‐Gli1. Gankyrin reduced Gli1 ubiquitination and induced Gli1 protein expression

As a chaperone of the ubiquitin‐proteasome, gankyrin can enhance the ubiquitylation and degradation of p53 by interacting with the E3 ubiquitin ligase MDM2.^29^ In addition, gankyrin can impede ubiquitination and degradation of the transcription factor Oct4 by the E3 ubiquitin ligase WWP2.^30^ We thus asked whether the up‐regulated Gli1 expression resulting from gankyrin amplification was controlled by ubiquitin‐mediated degradation. U2OS and MG63 cells were treated with the proteasome inhibitor MG132, and cellular lysates were immunoprecipitated with anti‐Gli1. As expected, gankyrin reduced Gli1 ubiquitination and induced Gli1 protein expression, indicating that gankyrin inhibits the ubiquitin‐mediated degradation of Gli1 protein (Figure 5A, B).

### 3.5 Gankyrin promotes the lung metastasis of OS in vivo

**FIGURE 6:**
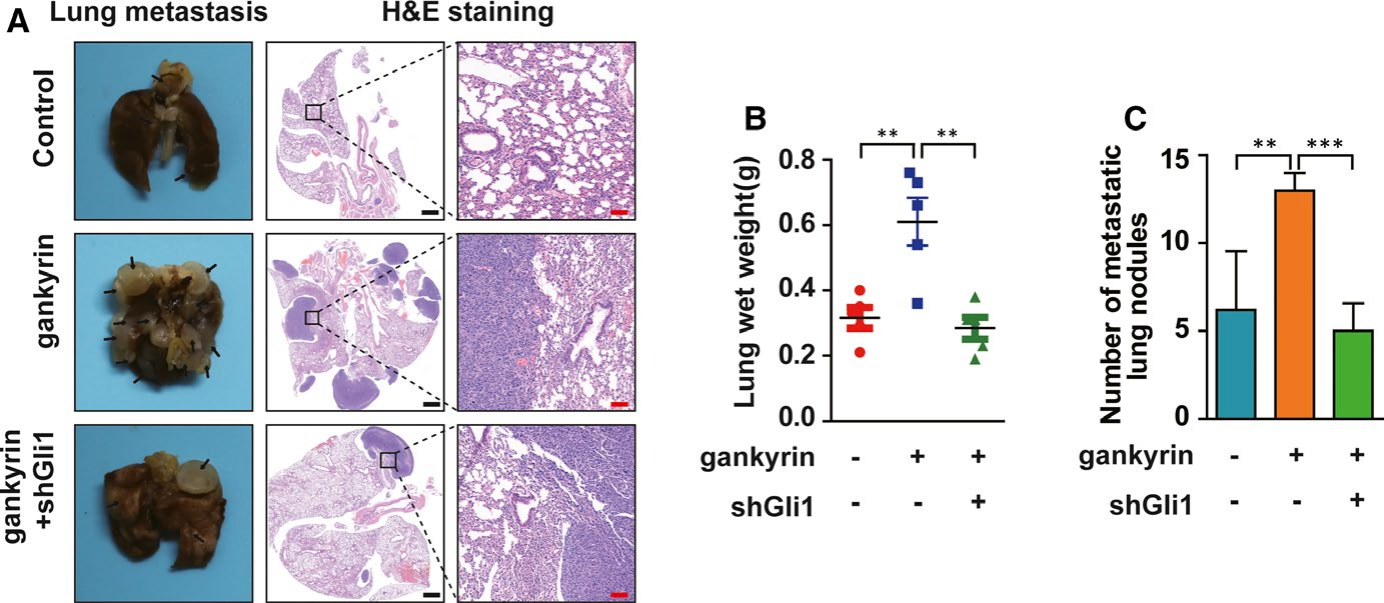
Gankyrin promotes the lung metastasis of OS in vivo. A, Overexpression of gankyrin promoted the metastasis of U2OS cells, and the increase in metastasis induced by gankyrin overexpression was attenuated through knockdown of Gli1. Representative images of metastatic lung nodules and H&E‐stained lung tissues are shown. Black scale bar, 1000 μm; red scale bar, 100 μm. B, Overexpression of gankyrin increased the lung weight, and knockdown of Gli1 abolished the increase in weight. **P < .01. C, Overexpression of gankyrin increased the number of metastatic lung nodules, and Gli1 knockdown abolished this increase. **P < .01; ***P < .001

Depending on the in vitro results, we have been suggested that gankyrin may induce lung metastasis of OS in vivo. Lung metastatic models of nude mice through tail vein injection with U2OS cells were established. Overexpression of gankyrin was shown to promote the lung metastasis of OS in vivo, as shown by increases in lung weight and the number of metastatic lung nodules. Furthermore, the increase in lung metastasis with gankyrin overexpression was attenuated through knockdown of Gli1 (Figure 6A‐C). Collectively, these data indicate that gankyrin promotes the lung metastasis of OS by regulating Gli1 in vivo.

## 4 DISCUSSION

**FIGURE 7:**
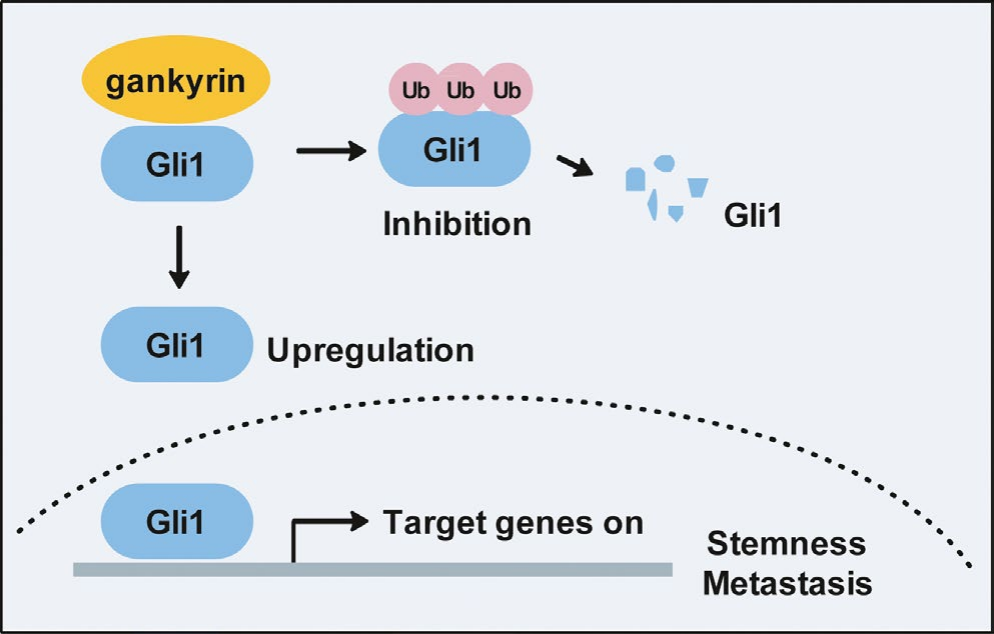
Proposed model for the mechanism by which the gankyrin/Gli1 axis promotes OS metastasis and regulates the expression of stemness factors

Previous studies have revealed that gankyrin regulates various signalling pathways in different cancers, such as mTORC1 in colorectal cancer,^31^ PI3K/AKT in ovarian cancer,^32^ RhoA/Rac1 in breast cancer^33^ and keap1/Nrf2 in hepatocellular carcinoma.^34^ In addition, our recent study identified gankyrin as a vital regulator of YAP in the tumorigenesis of OS.^5^ In this study, we initially found that gankyrin promotes migration, invasion and regulates the expression of some stemness factors by up‐regulating Gli1 in OS. Mechanistically, gankyrin binds with Gli1 and suppresses the ubiquitin‐mediated degradation of Gli1 protein. Importantly, gankyrin induces the lung metastasis of OS by regulating Gli1 in vivo. Our findings revealed a novel function and mechanism involving the oncoprotein gankyrin and Hedgehog signalling in OS progression (Figure 7A).

CSCs are usually more malignant than differentiated tumour cells and are involved in cancer invasion and metastasis.^9^, ^10^ Several previous studies have linked gankyrin to cancer stemness and metastasis.^23^, ^24^, ^35^ Gankyrin regulates stem cell behaviour by increasing stemness factor expression in colorectal cancer.^23^ Moreover, overexpression of gankyrin and stemness factor OCT4 promotes tamoxifen resistance in breast cancer.^24^ Consistent with these results, we observed enrichment of several CSC markers, including CD133, OCT4 and Nanog, via qRT‐PCR and Western blot analyses in OS cells. Unfortunately, the effect of gankyrin on the sphere formation ability of OS cells was not assessed in this study, because the technique used in our laboratory is incompletely developed. However, in summary, the qRT‐PCR and Western blot analyses can support our conclusion that gankyrin may affect the stemness property of OS cells. In addition to inducing OS stemness, gankyrin was found to promote metastasis in OS, which was demonstrated in vitro and in vivo (Figures 1, 2, 6). Combined with our previous discovery that gankyrin is tumorigenic in OS,^5^ these new findings lead us to speculate that gankyrin might be a potential molecular target in targeted therapy for OS.

The Hedgehog signalling pathway is considered a strong driver of the stemness and metastasis of various tumours.^17^, ^19^ Hence, it is of great significance to investigate the regulatory mechanisms of Hedgehog signalling. We observed in this study that gankyrin acts as an upstream regulatory molecule of the Hedgehog signalling Gli1 transcription factor in OS. Although our data support a positive correlation between gankyrin and Gli1 expression, it is unclear whether gankyrin directly interacts with Gli1 in OS because there was no suitable prokaryotic expression plasmid to perform GST pull‐down assays in our laboratory. Nonetheless, our discovery that gankyrin mediates the activation of Gli1 was confirmed. In addition, spatial and expression correlations have been demonstrated (Figure 4C‐F). The interaction if these proteins may also be relevant in several other tumours, such as colorectal cancer and breast cancer, as up‐regulation or activation of gankyrin and Gli1 has been shown in these tumours and overexpression of gankyrin or Gli1 appears to be a specific inducer of metastasis in these cancer types.^18^, ^33^, ^36^, ^37^

Another important finding in the present study is that gankyrin suppresses the ubiquitin‐mediated degradation of Gli1 protein in OS (Figure 5). Ubiquitylation is believed to be a complicated post‐translational modification process resulting in protein degradation.^38^ Ubiquitin‐mediated degradation is always regulated by an enzymatic cascade involving the E1 ubiquitin‐activating enzyme, E2 ubiquitin‐conjugating enzyme and E3 ubiquitin ligases and contributes to the transfer of ubiquitin molecules onto substrate proteins.^39^, ^40^ For instance, gankyrin interacts with the E3 ubiquitin ligase MDM2 to support the stability of P53.^29^ Gankyrin prevents the E3 ubiquitin ligase WWP2‐mediated degradation of Oct4.^30^ Despite our findings indicating a new role of gankyrin in mediating ubiquitination and degradation of Gli1, the precise enzymatic cascade is unexplored, and whether the E3 ubiquitin ligase MDM2 or WWP2 participates in this process is unclear. Regardless, our present study preliminarily discovered the potential mechanism by which gankyrin up‐regulates Gli1.

In summary, we highlighted gankyrin as an oncoprotein driving metastasis and regulating the expression of some stemness factors in OS. We showed a new positive association between gankyrin and Hedgehog/Gli1 signalling; thus, pharmacologic agents targeting the aberrant gankyrin/Gli1 axis may represent a promising therapeutic strategy for OS.

## CONFLICT OF INTEREST

The authors declare that they have no competing interests.

## AUTHOR CONTRIBUTIONS

**Chongchong Wang:** Data curation (equal); Formal analysis (equal); Funding acquisition (supporting); Writing‐original draft (lead). **Juehua Jing:** Conceptualization (equal); Data curation (equal); Formal analysis (equal). **Xuyang Hu:** Investigation (equal); Methodology (equal). **Shuisheng Yu:** Investigation (equal); Methodology (equal). **Fei Yao:** Investigation (equal); Methodology (equal). **Ziyu Li:** Formal analysis (equal). **Li Cheng:** Data curation (lead); Formal analysis (lead); Funding acquisition (lead); Project administration (lead); Writing‐review & editing (lead).

## ETHICAL APPROVAL AND CONSENT TO PARTICIPATE

Patients were recruited with a scientific ethics consent form, and all processes were approved by the Ethics Committee of the Second Affiliated Hospital of Anhui Medical University. All animal experiments were carried out according to the Guide for the Care and Use of Laboratory Animals and were approved by the Institutional Animal Care and Use Committee of Anhui Medical University.

## Supporting information

Fig S1

Fig S2

Table S1

## Data Availability

All data generated or analysed during this study are included in this published article.
