# Supplementary figures and images for: Gankyrin activates the hedgehog signalling to drive metastasis in osteosarcoma
Source: J Cell Mol Med. 2021 Jun 5;25(13):6232–41. doi: 10.1111/jcmm.16576 (PMC8366451; doi:10.1111/jcmm.16576)

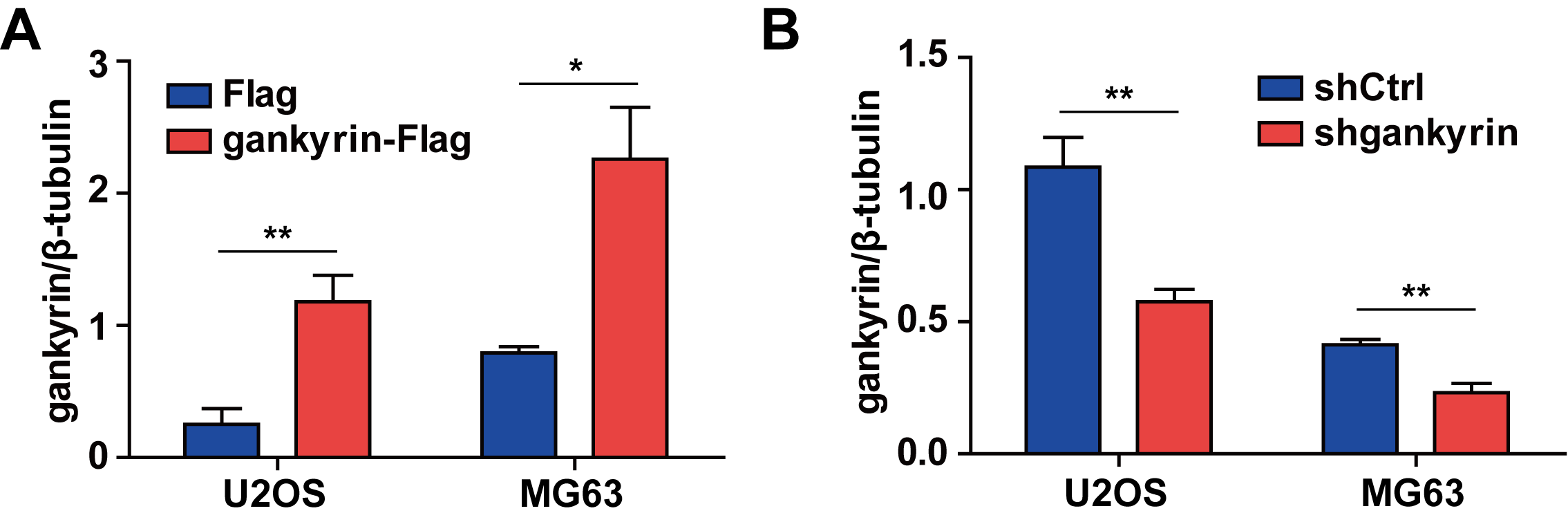

Supplement: Supplementary file 1 — Fig S1 [file JCMM-25-6232-s002.tif]

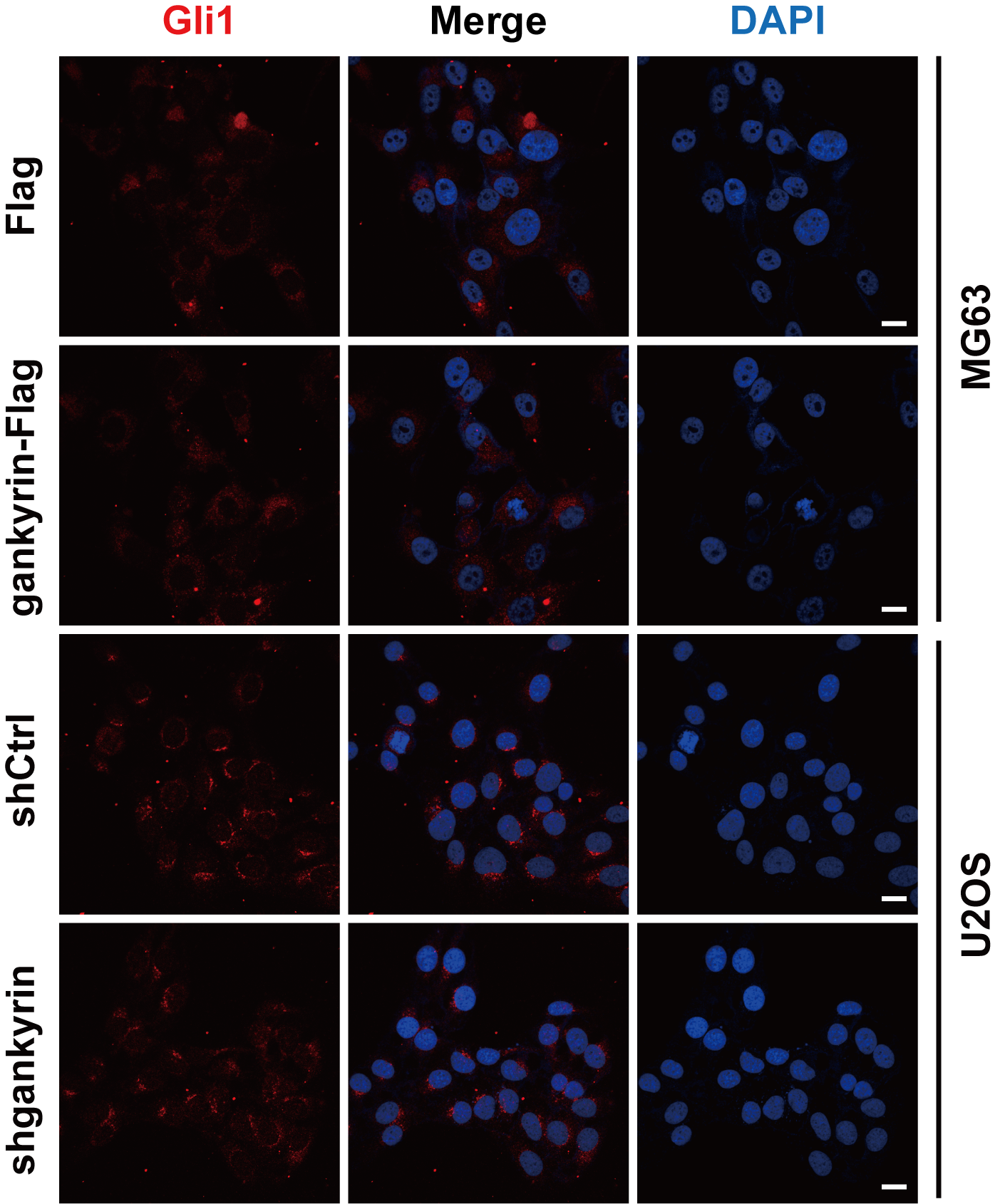

Supplement: Supplementary file 2 — Fig S2 [file JCMM-25-6232-s003.tif]
