# Supplementary material for: Gankyrin activates the hedgehog signalling to drive metastasis in osteosarcoma
Source: J Cell Mol Med. 2021 Jun 5;25(13):6232–41. doi: 10.1111/jcmm.16576 (PMC8366451; doi:10.1111/jcmm.16576)
Supplement: Supplementary file 3 — Table S1 [file JCMM-25-6232-s001.docx]

Supplementary table 1 Primer sequences

| Gene | Sense primer (5’-3’) | Antisense primer (5’-3’) |
| --- | --- | --- |
| CD133 | GCCACCGCTCTAGATACTGC | TGTTGTGATGGGCTTGTCAT |
| OCT4 | AGTGAGAGGCAACCTGGAGA | ACACTCGGACCACATCCTTC |
| Nanog | CAGTCTGGACACTGGCTGAA | CTCGCTGATTAGGCTCCAAC |
| Gli1 | CCACGGGGAGCGGAAGGAG | ACTGGCATTGCTGAAGGCTTTACTG |
| PTCH1 | CCCCTGTACGAAGTGGACACTC | AAGGAAGATCACCACTACCTTGG |
| β-catenin | GAAACGGCTTTCAGTTGAGC | CTGGCCATATCCACCAGAGT |
| AXIN2 | TCACCAAACCCATGTCTGTC | TCCAGGAAAGTTCGGAACAG |
| β-actin | ATCCACGAAACTACCTTCAACTCCAT | CATACTCCTGCTTGCTGATCCACATC |
